# Cyanogel-Derived Synthesis of Porous PdFe Nanohydrangeas as Electrocatalysts for Oxygen Reduction Reaction

**DOI:** 10.3390/nano11123382

**Published:** 2021-12-13

**Authors:** Jinxin Wan, Zhenyuan Liu, Xiaoyu Yang, Peng Cheng, Chao Yan

**Affiliations:** 1 School of Materials Science and Engineering, Jiangsu University of Science and Technology, Zhenjiang 212100, China; wjx192060036@163.com (J.W.); 15851702806@163.com (P.C.); 2 State Key Laboratory for Artificial Microstructure and Mesoscopic Physics, School of Physics, Peking University, Beijing 100871, China; yangxy1302@163.com

**Keywords:** PdFe alloy, cyanogel, porous nanohydrangeas, oxygen reduction reaction, fuel cells

## Abstract

It is important to develop cost-efficient electrocatalysts used in the oxygen reduction reaction (ORR) for widespread applications in fuel cells. Palladium (Pd) is a promising catalyst, due to its more abundant reserves and lower price than platinum (Pt), and doping an earth-abundant 3*d*-transition metal M into Pd to form Pd–M bimetallic alloys may not only further reduce the use of expensive Pd but also promote the electrocatalytic performance of ORR, owing to the synergistic effect between Pd and M. Here we report a cyanogel-derived synthesis of PdFe alloys with porous nanostructure via a simple coinstantaneous reduction reaction by using K_2_Pd^II^Cl_4_/K_4_Fe^II^(CN)_6_ cyanogel as precursor. The synthesized PdFe alloys possess hydrangea-like morphology and porous nanostructure, which are beneficial to the electrochemical performance in ORR. The onset potential of the porous PdFe nanohydrangeas is determined to be 0.988 V, which is much more positive than that of commercial Pt/C catalyst (0.976 V) and Pd black catalyst (0.964 V). Resulting from the unique structural advantages and synergetic effect between bimetals, the synthesized PdFe nanohydrangeas with porous structure have outstanding electrocatalytic activity and stability for ORR, compared with the commercial Pd black and Pt/C.

## 1. Introduction

Proton exchange membrane fuel cells (PEMFCs) have aroused increasing attention as one of the most promising renewable energy conversion systems on account of their high energy conversion efficiency, low operation temperature, and environmentally benign products [1,2,3]. As a crucial cathode reaction in PEMFCs, the oxygen reduction reaction (ORR) has attracted enormous research interest [4,5,6,7,8,9]. However, the overall efficiency of energy conversion is limited due to the intricate reaction process and inherently sluggish reaction kinetics of ORR. Generally, platinum (Pt) and Pt-based alloys have been deemed to be the most effective electrocatalysts for ORR [10,11,12]. Nevertheless, some decisive issues, including scarce reserve, high price, poor durability, and fuel crossover, hampered their large-scale commercialization for PEMFCs. In this connection, it is highly important to develop alternative Pt-free electrocatalysts with prominent catalytic properties in ORR. Palladium (Pd), in the same family as Pt, is more abundant and less expensive and has similar structure and properties to Pt and better electrocatalytic performance than Pt-based catalysts. Moreover, doping an earth-abundant 3*d*-transition metal M (M = Ni, Co, Cu, Fe, etc.) into Pd to form Pd–M bimetallic alloys can not only further reduce the use of expensive Pd but also tune the electronic structure of Pd effectively, which always results in excellent electrocatalytic performance [13,14,15,16]. For instance, Bharali’s group prepared carbon-supported PdNi electrocatalysts, which have remarkable ORR and formic acid oxidation behavior [17]. Cheng’s group demonstrated that one-dimensional PdCu nanocrystals prepared via a seed-mediated approach exhibited better performance for ORR [18]. Among varieties of 3*d*-transition metals, Fe has been recognized as a promising element to constitute Pd–Fe alloys because of its relatively low cost and elaborate synergistic effect [19,20,21]. The doping of Fe into a Pd lattice would shorten the interatomic distance of Pd–Pd and lead to compressive strain, which can quite improve the electrocatalytic activity of ORR by boosting the valid adsorption of active oxygen. What is more, because of the different electronegativities, the changing of the electronic structure for Pd with Fe results in the downshift of the Pd *d*-band center, which can efficiently reduce the adsorption of oxygenated species on the catalysts’ surface and increase the active sites. Therefore, PdFe nanoalloys are deemed to be greatly economical and efficient ORR electrocatalysts.

In addition, morphology control is another efficient method to affect the catalysts’ electrochemical properties. Porous nanostructures can enhance the atoms’ utilization efficiency of noble metals and possess a much larger surface area, providing abundant active sites, which can improve the activity [22,23,24]. What is more, the porous nanostructures not only can facilitate mass diffusion and transport but also hinder the dissolution and agglomeration of nanocatalysts. Hence, enormous efforts have been made to develop synthetic approaches to prepare Pd-based nanocatalysts with porous structures, including self-assembly [25], electrodeposition [26,27], hard template-engaged method [28,29], and so on. Despite these advances, those previously reported synthetic strategies are tedious and complex, dramatically impeding the large-scale practical applications of these nanomaterials in energy-related fields. Thus, it is greatly fulfilling to design a gentle and economical method for the preparation of Pd-based alloys with porous structure. Cyanogel is a category of coordinate polymer made from the reaction of a tetrachlorometalate ([RCl_4_]^2−^, R = Pt, Pd, Ir) and a transition metal cyanometalate ([M(CN)_n_]^2−/3−^, *n* = 4, 6; M = Fe, Co, Ni) in aqueous solution, as shown in Equation S1 (Supplementary Materials) [30,31,32,33]. Therefore, cyanogels can be used as various multi-metal precursors for the fabrication of bimetallic nanoalloys, avoiding the risk of alloy multiphases and complicated processes which frequently occur in the traditional reduction methods.

Herein, we demonstrate a simple preparation of PdFe nanohydrangeas with porous structure (PdFe PNHs) through a cyanogel-derived method at room temperature. The PdFe PNHs were obtained by using K_2_Pd^II^Cl_4_/K_4_Fe^II^(CN)_6_ cyanogel as precursor and hydrazine hydrate (N_2_H_4_·H_2_O) as reduction agent. Because of their hydrangea-like porous morphology and alloy nanostructure, the prepared PdFe PNHs have a high density of active sites. The electrochemically active surface area (ECSA) of the PdFe PNHs is 2.1 times larger than that of the commercial Pd black. Thus, the prepared PdFe PNHs exhibit enhanced electrochemical activity and durability for ORR in alkaline medium. Our strategy is simple, environmentally friendly, and will contribute to the further design of bimetallic alloys.

## 2. Materials and Methods

### 2.1. Reagents

Potassium tetrachloropalladite(II) (K_2_PdCl_4_), potassium hexacyanoferrate(II) (K_4_Fe(CN)_6_), and hydrazine hydrate (N_2_H_4_·H_2_O, 50%) were obtained from Sinopharm Chemical Reagent Co., Ltd. (Shanghai, China). Commercial Pd black and Pt/C were bought from Johnson Matthey Corporation. All chemicals and reagents were used without further purification. All of the aqueous solutions were prepared using Millipore water with a resistivity of 18.2 MΩ.

### 2.2. Synthesis of PdFe Porous Nanohydrangeas

In a typical synthesis, 6 mL of 50 mM K_2_PdCl_4_ and 3 mL of 50 mM K_4_Fe(CN)_6_ aqueous solutions were initially mixed at room temperature. After 5 min, the orange-red Pd–Fe cyanogel was formed. Then, 6 mL of diluted N_2_H_4_·H_2_O solution (containing 3 mL N_2_H_4_·H_2_O, 50%) was added into the Pd–Fe cyanogel slowly, and the resulting mixture was left to stand for an additional 36 h. After reaction, the black PdFe porous nanohydrangeas were separated by centrifugation at 15,000 rpm for 5 min, washed several times with water and NaOH solution, and then dried at 40 °C for 12 h in a vacuum oven.

### 2.3. Characterizations

The morphology and structure of products were measured with a Hitachi S-4800 scanning electron microscope (SEM) and JEOL JEM-2010 transmission electron microscope (TEM). High-resolution TEM (HRTEM), energy-dispersive X-ray (EDX), high-angle annular dark-field scanning transmission electron microscopy (HAADF-STEM), and elemental mapping measurements were carried out on a FEI Tecnai G2 F20 microscope, which was built as an accessory on the JEOL JEM-2100F. The Fourier transform infrared (FTIR) spectra were obtained with a Nicolet 520 SXFTIR spectrometer. The Brunauer–Emmett–Teller (BET) specific surface area and pore size distribution were examined at 77 K using a Micromeritics ASAP 2050 system. The phase purity and crystallinity of the products were confirmed by X-ray diffraction (XRD) on a Model D/max-rC X-ray diffractometer using Cu K*α* radiation source (*λ* = 1.5406 Å) and operating at 40 kV and 100 mA. X-ray photoelectron spectroscopy (XPS) tests were performed on a Thermo VG Scientific ESCALAB 250 spectrometer with a monochromatic Al K*α* X-ray source (1486.6 eV photons). The binding energy was trued with respect to C1s at 284.6 eV.

### 2.4. Electrochemical Measurements

All electrochemical experiments were performed on a CHI 760 D electrochemical workstation (CH Instruments, Chenhua Co., Shanghai, China,) at 25 °C. A standard three-electrode system was used for all electrochemical experiments which was made up of a platinum foil as the auxiliary electrode, a saturated calomel reference electrode as the reference electrode, and a RRDE modified with catalysts as the working electrode. Potentials in this work were recorded with respect to the reversible hydrogen electrode (RHE). The catalyst suspension was prepared by dispersing 5 mg of catalyst in 1 mL of solution containing 0.9 mL of deionized (DI) water and 0.1 mL of 0.5 wt% Nafion solution, followed by ultrasonication for 30 min. For the immobilization of catalyst, 10 μL of the catalyst suspension was dropped onto the RRDE surface (0.196 cm^2^) and then dried at ambient temperature. Cyclic voltammetry (CV) was performed in a N_2_-saturated 0.5 M H_2_SO_4_ solution. The ECSA of the catalysts could be calculated using the following equation: ECSA = *Q*/(*mC*), where *m* is the loading amount of Pd and the ECSA is calculated by integrating the charges (*Q*) associated with the peak from the reduction of Pd oxide, assuming 420 μC cm^−2^ is needed for the reduction of a Pd oxide monolayer. The ORR tests were carried out in an O_2_-saturated 0.1 M KOH solution with a sweep rate of 5 mV s^−1^ and a rotation rate of 1600 rpm. The electron transfer number (*n*) is calculated based on the equation of *n* = 4*I*_d_/(*I*_d_ + (*I*_r_/*N*)), where *I*_d_ and *I*_r_ stand for the disk current and ring current, respectively, and *N* is the current collection efficiency of the Pt ring (0.37).

## 3. Results and Discussion

**Figure 1:**
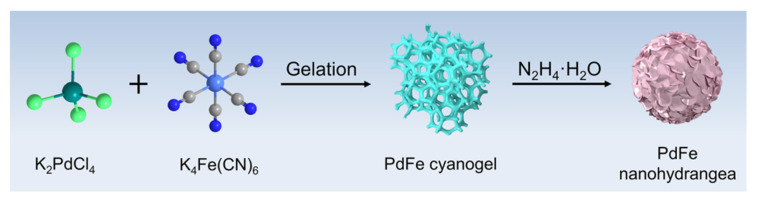
Schematic illustration of the formation of porous PdFe nanohydrangeas.

The overall synthesis process of PdFe nanohydrangeas is schematically shown in Figure 1. The orange-red PdFe cyanogels with three-dimensional (3D) network structure were achieved by mixing K_2_PdCl_4_ and K_4_Fe(CN)_6_ aqueous solutions (Figure S1). During the gelation process, the nitrogen end of the cyanide ligands in K_4_Fe(CN)_6_ could replace transchloride ligands in K_2_PdCl_4_. The characteristic stretching peak of C≡N has a positive shift relative to that of K_4_Fe(CN)_6_ in the FTIR spectrum (Figure S2), which validates the successful formation of PdFe cyanogels. After addition of N_2_H_4_·H_2_O to the cyanogels, the porous PdFe nanohydrangeas (PdFe PNHs) were obtained by centrifugation and washing.

**Figure 2:**
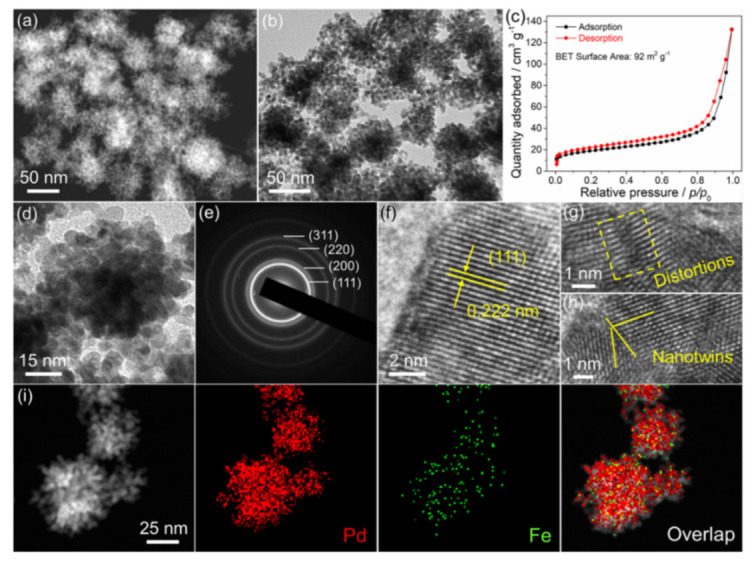
Morphological characterizations of the porous PdFe nanohydrangeas. (**a**) HAADF-STEM image; (**b**) TEM image; (**c**) N_2_ adsorption–desorption isotherms; (**d**) HRTEM; (**e**) SAED pattern; (**f**–**h**) magnified HRTEM images; (**i**) HAADF-STEM image and elemental mapping images.

The morphology and nanostructure of the PdFe PNHs were characterized with SEM, HAADF-STEM, and TEM. As viewed from SEM images (Figure S3) and the HAADF-STEM image (Figure 2a), the PdFe PNHs exhibit a porous, hydrangea-like structure. Consistent with the SEM and HAADF-STEM observation, the TEM image further confirms the porous, hydrangea-like nanostructure composed of interconnected small nanoparticles (Figure 2b). N_2_ adsorption–desorption isotherms of the PdFe PNHs display a distinctive hysteresis loop corresponding to type IV behavior (Figure 2c), suggesting that the porous structures are existent. Calculated from the N_2_ isotherms, the BET surface area is around 92 m^2^ g^−1^. The abundant pores and large surface area of the PdFe PNHs can facilitate transportation and infiltration of the reaction substances, offering more active surface sites, which benefit electrocatalytic reactions. The HRTEM image (Figure 2d) and the corresponding SAED pattern (Figure 2e) suggest that the PdFe PNHs consist of multiple coalesced crystalline segments with a face-centered cubic (*fcc*) polycrystalline structure. As marked in Figure 2f, the lattice plane with an interplanar distance of 0.222 nm is vested in the (111) plane in *fcc*-phased PdFe alloy. Furthermore, distortions and nanotwins can also be observed in the HRTEM images (Figure 2g,h); these can be used as active sites for electrocatalysis. Elemental mapping analysis discloses that Pd and Fe are distributed uniformly throughout the nanohydrangeas entirely, proving the formation of PdFe alloys (Figure 2i). This conclusion is further proved by the corresponding EDX line-scanning analysis (Figure S4), where Pd and Fe signals appear incidentally during the line-scanning process.

**Figure 3:**
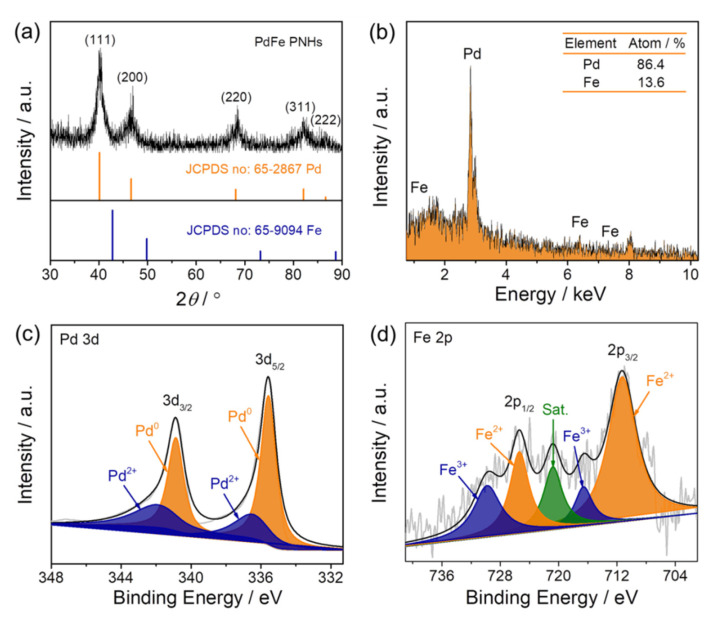
Compositional analyses of the porous PdFe nanohydrangeas. (**a**) XRD pattern; (**b**) EDX spectrum; (**c**) high-resolution Pd 3d XPS spectrum; (**d**) high-resolution Fe 2p XPS spectrum.

Figure 3a is the XRD pattern of the fabricated PdFe nanohydrangeas, which can be indexed to *fcc*-phased metal. In particular, all diffraction peaks are positioned between the standard peaks of pure Pd and Fe, proving the formation of PdFe alloys. As displayed in Figure 3b, the EDX spectrum implies the existence of Pd and Fe with a Pd/Fe molar ratio of 86.4:13.6 in the PdFe nanohydrangeas. As identified by XPS analysis (Figure S5), the Pd/Fe atomic ratio on the surface of the PdFe nanohydrangeas is 89.7:10.3, nearly identical to the data estimated by EDX. This result further demonstrates that Pd and Fe are homogeneously distributed throughout the PdFe nanohydrangeas. As exhibited in Figure 3c, the peaks located at 335.5 and 340.8 eV are vested in the metallic state of Pd in the PdFe catalysts, while the peaks at 336.5 and 341.9 eV are the oxidized species. The percentage of Pd^0^ species is approximately 84.9%, implying that Pd exists predominantly in a zero-valent state in the PdFe nanohydrangeas. Figure 3d is the high-resolution Fe 2p spectrum of the PdFe nanohydrangeas. According to previous reports [34,35,36], the peaks at 711.2 and 716.5 eV belong to the binding energies of 2p_3/2_ bands of Fe^2+^ and Fe^3+^ species, respectively. Meanwhile, the peaks at 725.3 and 729.7 eV are attributed to the binding energy of 2p_1/2_ orbitals of Fe^2+^ and Fe^3+^ species. The peak at 720.7 eV is a satellite peak for the above four peaks, indicating the coexistence of Fe^2+^ and Fe^3+^ in the catalyst.

**Figure 4:**
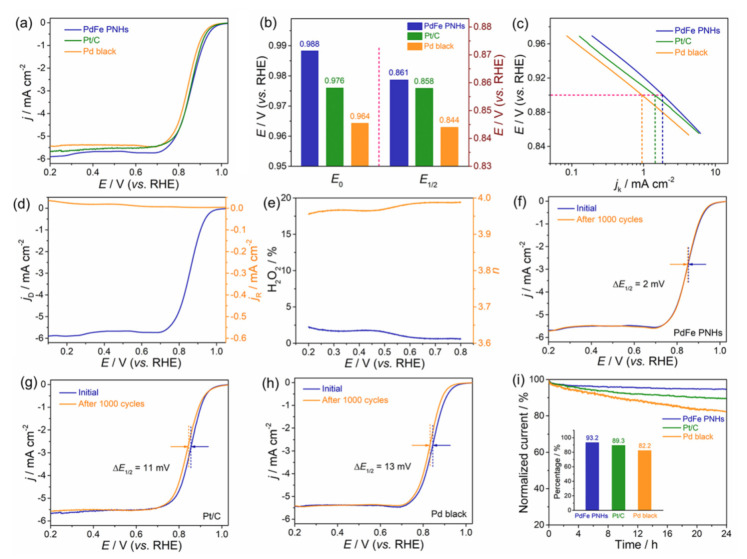
ORR performances of the porous PdFe nanohydrangeas, commercial Pd black, and Pt/C. (**a**) ORR polarization curves obtained in an O_2_-saturated 0.1 M KOH solution with a sweep rate of 5 mV s^−1^ and a rotation rate of 1600 rpm; (**b**) comparison of *E*_0_ and *E*_1/2_ for the three catalysts; (**c**) Tafel plots; (**d**) currents gathered on disk and ring electrodes catalyzed by the porous PdFe nanohydrangeas; (**e**) H_2_O_2_ yield and electron transfer number of the porous PdFe nanohydrangeas in ORR; (**f**) ORR polarization curves of the porous PdFe nanohydrangeas before and after 1000 cycles; (**g**) ORR polarization curves of the commercial Pt/C before and after 1000 cycles; (**h**) ORR polarization curves of the commercial Pd black before and after 1000 cycles; (**i**) CA tests of the three catalysts in O_2_-saturated 0.1 M KOH.

In consideration of the picturesque porous, hydrangea-like morphology and alloy nanostructure, the synthesized PdFe PNHs are supposed to have excellent electrocatalytic performance. Accordingly, the electrocatalytic properties of the porous PdFe nanohydrangeas for ORR were measured in alkaline medium to emphasize their structural and compositional advantages. For comparison, commercial Pd black and Pt/C catalysts were also appraised under the same experimental conditions. Figure S6a shows the representative CV curves of the two electrocatalysts in the N_2_-saturated 0.5 M H_2_SO_4_ solution. According to the integrated charges for the reduction of PdO monolayer during the cathodic scan [37,38], the ECSA of the porous PdFe nanohydrangeas is calculated to be 15.5 m^2^ g^−1^, while that of the commercial Pd black is 7.4 m^2^ g^−1^. The prominent enhancement of the ECSA of the PdFe PNHs may result from its porous characteristics, with large surface area. Firstly, we tested the ORR performance of the three catalysts in O_2_-saturated 0.1 M KOH solution with a sweep rate of 5 mV s^−1^ and a rotation rate of 1600 rpm. Figure 4a shows the typical ORR polarization curves of the three catalysts. As summarized in Figure 4b, the onset potential (*E*_0_) and half-wave potential (*E*_1/2_) of the porous PdFe nanohydrangeas are determined to be 0.988 and 0.861 V, respectively, which are much more positive than those of commercial Pt/C catalyst (0.976 and 0.858 V) and Pd black catalyst (0.964 and 0.844 V) and able to be compared with those of the reported electrocatalysts (Table S1). Figure 4c shows the corresponding Tafel plots of the kinetic current density (*j*_k_) computed from the ORR polarization curves. Notably, the *j*_k_ value of the PdFe PNHs is the highest at the same applied potential, indicating that the PdFe PNHs possess better kinetic behavior than commercial Pd black and Pt/C catalysts. Together with the more positive *E*_0_ and *E*_1/2_ data, these consequences forcefully show that the porous PdFe nanohydrangeas exceed the reference catalysts with respect to ORR. To further study the ORR mechanism of the porous PdFe nanohydrangeas, we used RRDE tests to trace the production yield of hyperoxide species (HO_2_^-^) during the ORR process (Figure 4d). Figure 4e suggests that the peroxide yield is quite low (<5%) for the PdFe PNHs during ORR. The number of electron transfers (*n*) is calculated to be 3.95–3.99 at the potential of 0.2–0.8 V, revealing that the ORR process is a four-electron pathway. Moreover, the electron transfer number can also be calculated from the Koutecky–Levich (K–L) plots. Figure S7 displays the ORR polarization curves of the three catalysts obtained in O_2_-saturated 0.1 M KOH solution with a sweep rate of 5 mV s^−1^ and different rotation speeds, as well as the relevant K–L plots. As expected, the PdFe PNHs shows a high electron transfer number (3.96) which is comparable to those of commercial Pt/C (3.92) and Pd black (3.89) catalysts.

In addition, long-term stability is also a significant aspect of electrocatalysts to evaluate electrocatalytic performance. Accelerated durability tests (ADTs) for 1000 cycles were carried out to evaluate the ORR durability of the three catalysts (Figure 4f–h). The half-wave potential of the PdFe PNHs exhibits an unspectacular degradation with just 2 mV negative shift, which is much lower than the commercial Pt/C (11 mV) and Pd black (13 mV). Furthermore, the stability of the three catalysts was also tested by chronoamperometry (CA) measured in O_2_-saturated 0.1 M KOH for 24 h. As displayed in Figure 4i, the current intensity decay of PdFe PNHs is much lower than that of the commercial Pt/C and Pd black during the entire time range. The PdFe PNHs still maintain 93.2% of original current density after tests, whereas the current losses of commercial Pt/C and Pd black are 10.7% and 17.8% (inset of Figure 4i), respectively, implying that the PdFe PNHs have better stability as electrocatalysts of ORR. The outstanding ORR activity and stability of the PdFe PNHs can be put down to the unique porous, hydrangea-like morphology and alloyed components. Concretely, (i) the unique porous, hydrangea-like structure composed of interconnected small nanoparticles has a large surface area and abundant accessible active sites, which can boost mass diffusion and transport during the electrochemical course and eventually promote the reaction kinetics; (ii) the synergistic effects between Pd and Fe elements increase the efficient adsorption of active oxygen and migration of rival hydroxyl species on the surface of the PdFe PNHs, thus promoting the direct four-electron transfer and resulting in a superior ORR performance [39,40].

## 4. Conclusions

In conclusion, we have presented a cyanogel-derived synthetic method for the fabrication of the porous PdFe nanohydrangeas. This strategy contains the formation of PdFe cyanogel and then chemical reduction with N_2_H_4_·H_2_O solution. The as-synthesized PdFe alloys have unique porous, hydrangea-like structure composed of interconnected small nanoparticles. Because of the favorable structural advantages and synergistic effects between Pd and Fe, the porous PdFe nanohydrangeas show excellent electrocatalytic activity and stability compared with the commercial Pd black and Pt/C catalysts with respect to ORR. This work offers a simple method for the development of Pd-based alloy nanocatalysts with porous structure which could advance the widespread applications of PEMFCs.

## Data Availability

Data are contained within the article.

## Supplementary Materials

The following are available online at https://www.mdpi.com/article/10.3390/nano11123382/s1, Equation S1: Formation equation of cyanogel from transition metal cyanometalates and tetrachlorometalates in aqueous solution, Figure S1: Digital photos showing the formation of PdFe cyanogel, Figure S2: FTIR spectra of (a) the K_2_PdCl_4_/K_4_Fe(CN)_6_ cyanogel and (b) pure K_4_Fe(CN)_6_, Figure S3: Typical SEM images of the porous PdFe nanohydrangeas at different magnifications, Figure S4: (a) STEM image and (b) EDX line scanning profile of the porous PdFe nanohydrangeas, Figure S5: XPS survey scan spectrum of the porous PdFe nanohydrangeas, Figure S6: (a) CV curves of the as-synthesized porous PdFe nanohydrangeas and commercial Pd black catalyst recorded in N2-purged 0.5 M H2SO4 solution with a sweep rate of 50 mV s^−1^ and (b) Graphical comparison of the ECSA of the two catalysts, Figure S7: ORR polarization curves of the three catalysts obtained in O2-saturated 0.1 M KOH solution with a sweep rate of 5 mV s^−1^ and different rotation speeds. (a) PdFe PNHs; (b) Commercial Pt/C; (c) Commercial Pd black. The relevant Koutecky-Levich plots at different potentials of the three catalysts. (d) PdFe PNHs; (e) Commercial Pt/C; (f) Commercial Pd black, Table S1: Comparison of the ORR activity of the porous PdFe nanohydrangeas with other electrocatalysts previously reported. References [41,42,43,44,45,46,47,48] were cited in Supplementary Materials.
