# Supplementary material for: Cyanogel-Derived Synthesis of Porous PdFe Nanohydrangeas as Electrocatalysts for Oxygen Reduction Reaction
Source: Nanomaterials (Basel). 2021 Dec 13;11(12):3382. doi: 10.3390/nano11123382 (PMC8708350; doi:10.3390/nano11123382)
Supplement: Supplementary file 1 [file nanomaterials-11-03382-s001.zip › nanomaterials-1478497-supplementary.pdf]

Supplementary Materials

Cyanogel-Derived Synthesis of Porous PdFe Nanohydrangeas as Electrocatalysts for Oxygen Reduction Reaction

Jinxin Wan [1,†], Zhenyuan Liu [1,*,†], Xiaoyu Yang [2,†], Peng Cheng [1] and Chao Yan [1,*]

[1] School of Materials Science and Engineering, Jiangsu University of Science and Technology, Zhenjiang 212100, China; wjx192060036@163.com (J.W.); 15851702806@163.com (P.C.)
[2] State Key Laboratory for Artificial Microstructure and Mesoscopic Physics, School of Physics, Peking University, Beijing 100871, China; yangxy1302@163.com
* Correspondence: zhenyuanliu@just.edu.cn (Z.L.); chaoyan@just.edu.cn (C.Y.)
† These authors contributed equally to this paper.

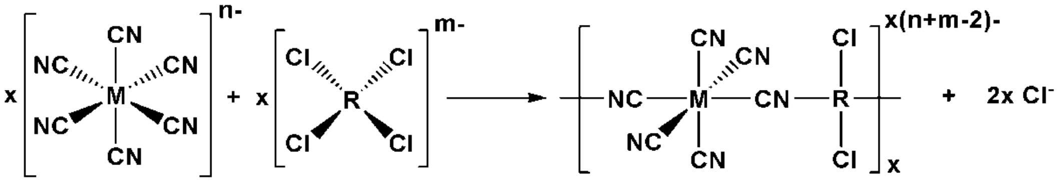

Equation S1. Formation equation of cyanogel from transition metal cyanometalates and tetrachlorometalates in aqueous solution.

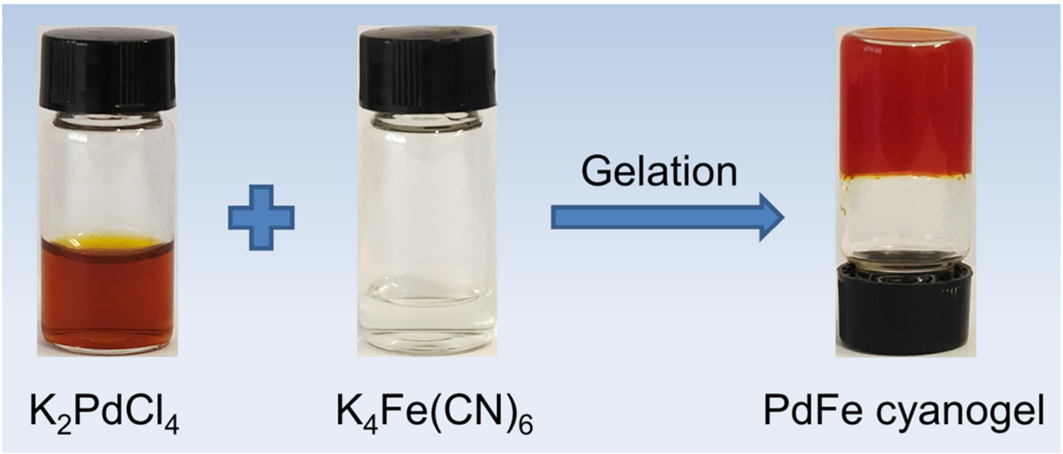

Figure S1. Digital photos showing the formation of PdFe cyanogel.

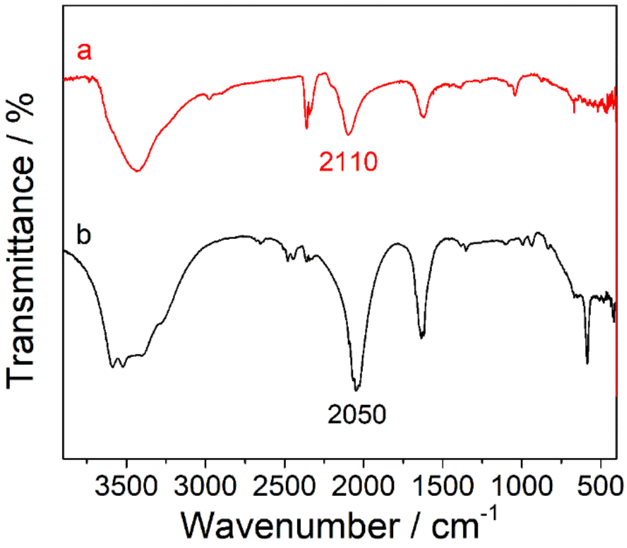

Figure S2. FTIR spectra of (a) the K2PdCl4/K4Fe(CN)6 cyanogel and (b) pure K4Fe(CN)6.

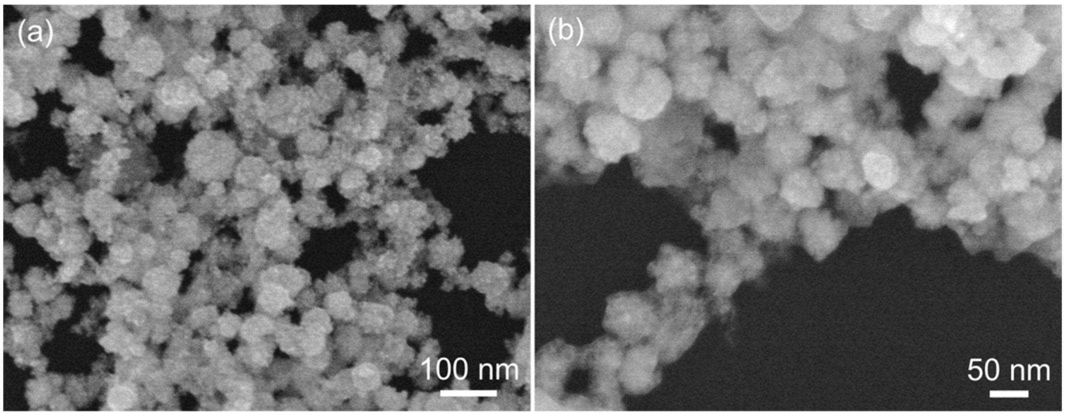

Figure S3. Typical SEM images of the porous PdFe nanohydrangeas at different magnifications.

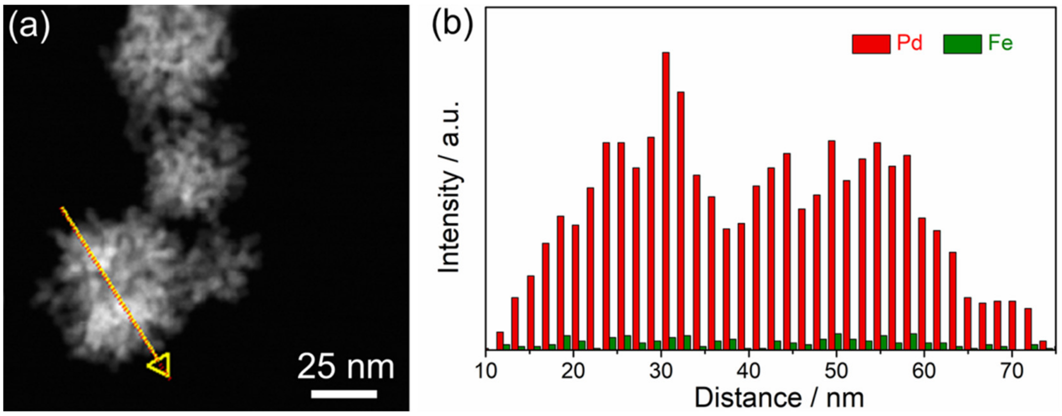

Figure S4. (**a**) STEM image and (**b**) EDX line scanning profile of the porous PdFe nanohydrangeas.

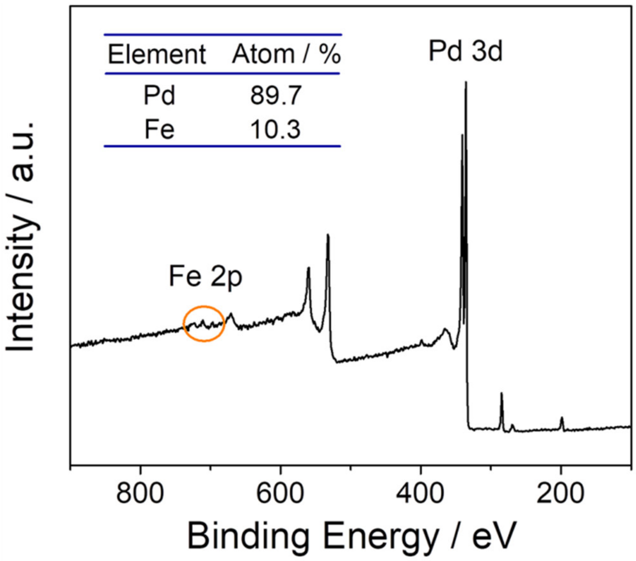

Figure S5. XPS survey scan spectrum of the porous PdFe nanohydrangeas.

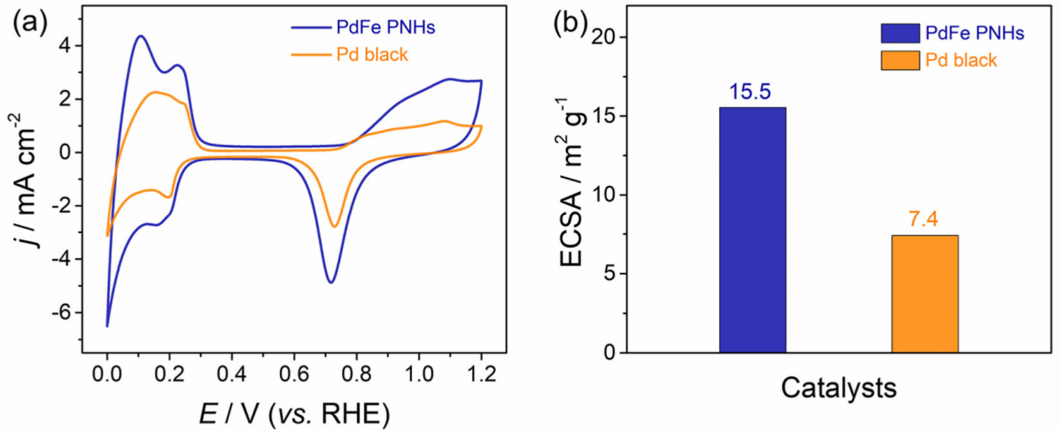

Figure S6. (**a**) CV curves of the as-synthesized porous PdFe nanohydrangeas and commercial Pd black catalyst recorded in N_2-purged 0.5 M H_2SO_4 solution with a sweep rate of 50 mV s^{-1} and (**b**) Graphical comparison of the ECSA of the two catalysts.

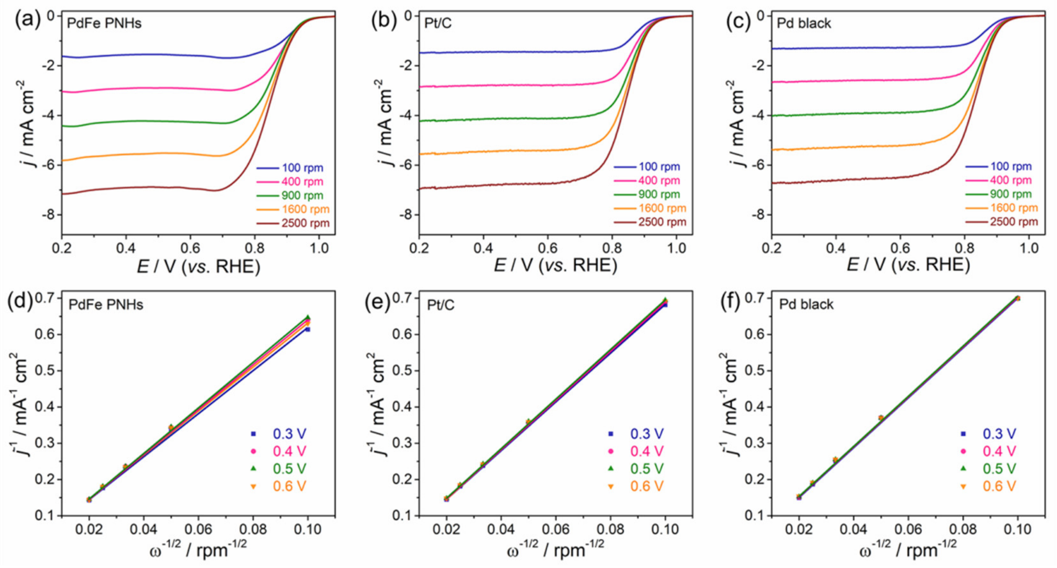

Figure S7. ORR polarization curves of the three catalysts obtained in O2-saturated 0.1 M KOH solution with a sweep rate of 5 mV s^{-1} and different rotation speeds. (**a**) PdFe PNHs; (**b**) Commercial Pt/C; (**c**) Commercial Pd black. The relevant Koutecky-Levich plots at different potentials of the three catalysts. (**d**) PdFe PNHs; (**e**) Commercial Pt/C; (**f**) Commercial Pd black.

Table S1. Comparison **of** the ORR activity of the porous PdFe nanohydrangeas with other electrocatalysts previously reported.

Catalyst	E_0 / V	$E_{1/2}$ / V	Electrolyte	Ref
**PdFe PNHs**	**0.988**	**0.861**	**0.1 M KOH**	**this work**
**Pt/C**	**0.976**	**0.858**	**0.1 M KOH**	**this work**
**Pd black**	**0.964**	**0.844**	**0.1 M KOH**	**this work**
Pd NPAs	0.926	0.837	0.1 M KOH	[1]
Pt_2Pd/NPG	0.97	0.84	0.1 M KOH	[2]
Pd_3Ni/C	0.96	0.85	0.1 M KOH	[3]
Pd_3Cu/C	0.875	0.785	0.1 M KOH	[4]
PdZn	0.98	0.82	0.1 M KOH	[5]
NiPd NPs/N-GR	0.961	0.82	0.1 M KOH	[6]
PdCu NTs	0.941	0.824	0.1 M KOH	[7]
$MnPd_3$/C	0.953	0.80	0.1 M KOH	[8]
Pd_2NiAg	0.923	0.842	0.1 M KOH	[9]
PdCo/NPC	0.928	0.843	0.1 M KOH	[10]

References

1. Liu, S.L.; Mu, X.Q.; Duan, H.Y.; Chen, C.Y.; Zhang, H. Pd Nanoparticle Assemblies as Efficient Catalysts for the Hydrogen Evolution and Oxygen Reduction Reactions. *European Journal of Inorganic Chemistry* **2017**, *2017*, 535-539.
2. Zhong, X.; Qin, Y.Y.; Chen, X.L.; Xu, W.L.; Zhuang, G.; Li, X.N.; Wang, J.G. PtPd alloy embedded in nitrogen-rich graphene nanopores: High-performance bifunctional electrocatalysts for hydrogen evolution and oxygen reduction. *Carbon* **2017**, *114*, 740-748.
3. Goswami, C.; Saikia, H.; Tada, K.; Tanaka, S.; Sudarsanam, P.; Bhargava, S.K.; Bharali, P. Bimetallic Palladium–Nickel Nanoparticles Anchored on Carbon as High-Performance Electrocatalysts for Oxygen Reduction and Formic Acid Oxidation Reactions. *ACS Applied Energy Materials* **2020**, *3*, 9285-9295.

4. Goswami, C.; Saikia, H.; Jyoti Borah, B.; Jyoti Kalita, M.; Tada, K.; Tanaka, S.; Bharali, P. Boosting the electrocatalytic activity of Pd/C by Cu alloying: Insight on Pd/Cu composition and reaction pathway. *Journal of Colloid and Interface Science* **2021**, *587*, 446-456.
5. Yang, H.Y.; Wang, K.; Tang, Z.H.; Liu, Z.; Chen, S.W. Bimetallic PdZn nanoparticles for oxygen reduction reaction in alkaline medium: The effects of surface structure. *Journal of Catalysis* **2020**, *382*, 181-191.
6. Thi, M.L.N.; Tran, T.H.; Anh, P.D.H.; Nhac-Vu, H.T.; Bui, Q.B. An innovative catalyst of nickel-palladium alloy nanocrystals embedded nitrogen-doped graphene for efficient oxygen reduction reaction. *Journal of Alloys and Compounds* **2019**, *797*, 314-324.
7. Wu, D.F.; Cheng, D.J. Structure-controlled synthesis of one-dimensional PdCu nanoscatalysts via a seed-mediated approach for oxygen reduction reaction. *Applied Surface Science* **2019**, *493*, 139-145.
8. Lu, Y.N.; Zhao, S.L.; Yang, R.; Xu, D.D.; Yang, J.; Lin, Y.; Shi, N.E.; Dai, Z.H.; Bao, J.C.; Han, M. Well-Coupled Nanohybrids Obtained by Component-Controlled Synthesis and in Situ Integration of Mn_xPd_y Nanocrystals on Vulcan Carbon for Electrocatalytic Oxygen Reduction. *ACS Applied Materials & Interfaces* **2018**, *10*, 8155-8164.
9. Liu, S.L.; Zhang, Q.H.; Li, Y.F.; Han, M.; Gu, L.; Nan, C.W.; Bao, J.C.; Dai, Z.H. Five-Fold Twinned Pd_2NiAg Nanocrystals with Increased Surface Ni Site Availability to Improve Oxygen Reduction Activity. *Journal of the American Chemical Society* **2015**, *137*, 2820-2823.
10. Xue, H.R.; Tang, J.; Gong, H.; Guo, H.; Fan, X.L.; Wang, T.; He, J.P.; Yamauchi, Y. Fabrication of PdCo Bimetallic Nanoparticles Anchored on Three-Dimensional Ordered N-Doped Porous Carbon as an Efficient Catalyst for Oxygen Reduction Reaction. *ACS Applied Materials & Interfaces* **2016**, *8*, 20766-20771.
